# Epidemiology of hand, foot, and mouth disease and the genetic characteristics of Coxsackievirus A16 in Taiyuan, Shanxi, China from 2010 to 2021

**DOI:** 10.3389/fcimb.2022.1040414

**Published:** 2022-11-11

**Authors:** Jiane Guo, Zijun Cao, Hongyan Liu, Jihong Xu, Lifeng Zhao, Li Gao, Zhihong Zuo, Yang Song, Zhenzhi Han, Yong Zhang, Jitao Wang

**Affiliations:** ^1^ School of Public Health, Shanxi Medical University, Taiyuan, Shanxi, China; ^2^ Department of Microbiology Test, Taiyuan Center for Disease Control and Prevention, Taiyuan, Shanxi, China; ^3^ World Health Organization (WHO) Western Pacific Region Office (WPRO) Regional Polio Reference Laboratory, National Health Commission Key Laboratory of Biosafety, National Health Commission Key Laboratory of Medical Virology, National Institute for Viral Disease Control and Prevention, Chinese Center for Disease Control and Prevention, Beijing, China; ^4^ Center for Biosafety Mega-Science, Chinese Academy of Sciences, Wuhan, China

**Keywords:** hand, foot, and mouth disease, epidemiology, Coxsackievirus A16, genetic characteristics, *VP1*

## Abstract

Hand, foot, and mouth disease (HFMD) is a common childhood infectious disease caused by human enteroviruses (EV). This study aimed to describe the epidemiological features of HFMD and the genetic characteristics of Coxsackievirus A16 (CVA16) in Taiyuan, Shanxi, China, from 2010 to 2021. Descriptive epidemiological methods were used to analyze the time and population distribution of HFMD and the genetic characteristics of CVA16. Except being affected by the COVID-19 epidemic in 2020, HFMD epidemics were sporadic from January to March each year, and began to increase in April, with a major epidemic peak from May to August, which declined in September, followed by a secondary peak from October to December. The prevalence of EV infection was the highest in children aged one to five years (84.42%), whereas its incidence was very low in children under one year of age (5.48%). Enterovirus nucleic acid was detected by real-time reverse transcription polymerase chain reaction in 6641 clinical specimens collected from patients with HFMD from 2010 to 2021, and 4236 EV-positive specimens were detected, including 988 enterovirus A71 (EV-A71), 1488 CVA16, and 1760 other enteroviruses. CVA16 remains prevalent and has co-circulated with other EVs in Taiyuan from 2010 to 2021. A phylogenetic tree constructed based on the *VP1* region showed that all CVA16 strains belonged to two different clades of the B1 genotype, B1a and B1b. They showed a nucleotide similarity of 86.5–100%, and an amino acid similarity of 96.9–100%. Overall, these findings add to the global genetic resources of CVA16, demonstrate the epidemiological characteristics of HFMD as well as the genetic features of CVA16 in Taiyuan City during 2010–2021, and provide supporting evidence for the prevention and control of HFMD.

## Introduction

Hand, foot, and mouth disease (HFMD) was first described in New Zealand in 1957 and is a common childhood infectious illness (Nhu et al., 2021). It is prevalent worldwide and occurs mainly in children under 5 years of age (Xing et al., 2014; Ji et al., 2019). In the past decades, particularly in the Asia-Pacific region, multiple outbreaks of HFMD have been recorded in Singapore, Vietnam, and mainland China (Lim et al., 2016; Cai et al., 2019; Van et al., 2019). After several large HFMD outbreaks during 2007 and early 2008, HFMD was defined as a C-class notifiable communicable disease by the Ministry of Health of China and an HFMD virological surveillance system was set up in 2008 (Zhang et al., 2009; Tian et al., 2014). HFMD is associated with fever and/or typical vesicular rashes on the hands, feet, buttocks, or mouth as the main symptoms. Patients with HFMD mostly develop mild and self-limited symptoms, but may also progress to serious illnesses such as acute flaccid paralysis, encephalitis, myocarditis, and encephalomyelitis (Li et al., 2005; Esposito and Principi, 2018; Yin et al., 2018).

HFMD is caused by human enterovirus (EV), a non-enveloped single positive-stranded RNA virus with a total genome length of about 7.4 kb, which encodes four structural proteins (VP1-VP4); the VP1 region is commonly used for enterovirus genotyping (Noisumdaeng et al., 2019). EVs can be divided into four species (A-D). Most HFMD cases are caused by the species EV-A, which includes two major pathogens, coxsackievirus A16 (CVA16) and enterovirus A71 (EV-A71) (Zhang and Xu, 2013). In recent years, national surveillance of HFMD cases has revealed that the pathogens causing HFMD are constantly changing. After applying the EV-A71 inactivated vaccine marketed in China in 2016, the number of HFMD caused by EV-A71 has decreased rapidly (Hong et al., 2022), whereas the proportion of CVA16 has gradually increased, and the proportion of other EVs in the pathogen spectrum has increased significantly (Chen et al., 2017; He et al., 2018). This indicates that the monovalent EV-A71 inactivated vaccine can prevent 90% of EV-A71-induced HFMD cases, but provides no cross-protection against infection with CVA16 and other EVs (Li et al., 2014; Li et al., 2016; Takahashi et al., 2016), resulting in the consistently high number of CVA16-related HFMD cases.

HFMD, with the exception of Polar regions, is global in distribution (Cox et al., 2017). It has been prevalent since the 1990’s and has subsequently caused large-scale epidemics every few years with notified cases exceeding a million each year (Koh et al., 2018). In China, as of December 2021, the cumulative total number of reported cases reached approximately 24.57 million since HFMD was listed as a notifiable infectious disease in 2008, 3698 of whom died according to the National Health Commission of the People’s Republic of China (http://www.nhc.gov.cn/).

Shanxi Province is located in central China and its capital city, Taiyuan, is located at the center of Shanxi Province. Taiyuan City established a HFMD surveillance system in 2010, but the epidemic and genetic characteristics of CVA16 remain unclear, and especially require continuous surveillance and analysis. To address this problem, we investigated the epidemiology of hand, foot, and mouth disease and the genetic characteristics of Coxsackievirus A16 in Taiyuan, Shanxi, China from 2010 to 2021.

## Methods

### Case definitions

HFMD case-reporting criteria are defined in the national guidelines for control and prevention for HFMD (issued by Ministry of Health in China (2009)). For clinical diagnosis, HFMD cases include mild and severe cases. Mild cases are defined as fever or not showing fever accompanied by rash (maculopapular or vesicular rash) appearing at the sites of hand, foot, mouth or buttock. Severe cases displayed severe neurological or cardiopulmonary clinical symptoms, such as acute flaccid paralysis, aseptic meningitis and pulmonary hemorrhage.

### Epidemiological analysis

The demographic data were obtained from the statistical yearbook of Shanxi Province. The data of HFMD cases in Taiyuan City from January 2010 to December 2021 was obtained from the China Information System for Disease Control and Prevention. The data of the reported cases included the gender, age, and onset of the disease time, which were sorted using Excel software. Statistical analyses were performed to describe epidemiological features, including demographic characteristics, gender and age distribution and seasonal variation. Total incidence was defined as the total number of HFMD cases divided by the average population size during the study period. Data analyses were performed using SPSS Statistics software (Version 26.0). Chi-square was performed on the patients’ demographic categories. Statistical significance was set at P < 0.05.

### Specimen collection

Throat swab or stool specimens were collected from clinically diagnosed HFMD cases within three days of onset at the district and county Centers for Disease Control and Prevention (CDC) in Taiyuan City. In total, 6641 specimens were collected during 2010–2021 and sent to the virus microbiology laboratory in Taiyuan CDC for enterovirus nucleic acid detection. Upon receipt at the laboratory, each specimen was assigned a unique laboratory code and entered into the Gastroenteritis Information Database at Taiyuan CDC.

### RNA extraction

According to the manufacturer’s instructions, total RNA was extracted from 50 μL of clinical specimens using a MagMax-96 Viral RNA Isolation Kit (Thermo Fisher

Scientific, Foster City, CA). RNA-positive control and negative control (ddH_2_O) was included in the extraction procedure in each batch. The step of RNA extraction was done in a biosafety cabinet.

### EV identification

RNA samples were tested for EV typing using Enterovirus 71 & Coxsackie Virus A16 & Enterovirus Real Time RT-PCR Kit (Shanghai ZJ Bio-Tech Co., Ltd., Shanghai, China) and CFX96™ Real-time PCR Detection System (CFX96, Bio-Rad, Hercules, California, USA).

A 25 μL reaction system comprising 1 μL of enzyme mixture,19 μL of reaction solution, and 5 μL of viral nucleic acid was prepared. PCR cycling parameters were set up according to the manufacturer’s instructions as follows: 50°C for 30 min, 95°C for 10 min, followed by 45 cycles of 95°C for 10 s, and 55°C for 40 s. A positive result was defined as a cycle threshold (Ct) value ≤ 43, and the positive control was defined as a Ct value ≤ 35.

### *VP1* region amplification and nucleotide sequencing

In total, 362 specimens were randomly selected to amplify the *VP1* region with specific primer pairs for CVA16 (upstream primer, CVA16-VP1-S: ATTGGTGCTCCCACTACAGC, and downstream primer, CVA16-VP1-A: GCTGTCCTCCCACACAAGAT) (Zhang et al., 2010). The primers yielded a CVA16 amplification product of 1110 bp, spanning the entire *VP1* sequence (891 nucleotides). A PrimeScript One Step RT-PCR Kit (TaKaRa, Dalian, China) was used for amplifying the *VP1* region. The amplification conditions were as follows: reverse transcription at 50°C for 30 min, initial denaturation at 94°C for 2 min, followed by 32 cycles of denaturation at 94°C for 30 s, annealing at 50°C for 30 s, extension at 72°C for 80 s, and final extension at 72°C for 10 min to complete the amplification. All the PCR runs included the positive control and negative control to avoid false-positive results. All the primers were synthesized by Sangon Biotech (Shanghai, China). The amplified products (5 μL) were electrophoresed through 1.0% agarose gel in Tris-acetate-EDTA buffer and were identified according to the position corresponding to DNA molecular weight standards (Marker). The PCR products were purified by gel extraction and sequenced by Ruiboxingke Biotechnology Co., Ltd. (Beijing, China).

### Phylogenetic analysis and determination of CVA16 genotypes

The resulting sequences were spliced and aligned using Sequencher 5.4.6 software; nucleotide and amino acid similarity analyses were performed using BioEdit software (version 7.2.5). The entire *VP1* sequences of Taiyuan CVA16 strains were aligned with the reference strain worldwide using pairwise alignment in MEGA (version 11.0.11). A phylogenetic tree was constructed using the neighbor-joining method, and the reliability of the tree was evaluated using 1000 bootstrap replicates (Tamura et al., 2021). The reference sequences used for homology analysis and phylogenetic tree construction were obtained from the GenBank database of the National Center for Biotechnology Information. The reference sequences are shown in **Table 1**.

**Table 1:** Reference sequences used for plotting the phylogenetic tree in this study.

Strains	Accession number	Year	Location	Genotype
G10	U05876	1951	South Africa	A
CVA-16/11/CHN/GZ/2018	MT119437	2018	Guangdong	B1a
CA16/TH/MUMT-1/2017	MH879032	2017	Thailand	B1a
CVA16/HVN17.120/Hai_Phong/VNM/2017	LC438268	2017	Vietnam	B1a
2014-YT309-CVA16(B1a)	KX586352	2014	Shandong	B1a
S701/BJ/CHN/2010	MT553119	2010	Beijing	B1a
1575-Yamagata-2010	AB634448	2010	Japan	B1a
GS0363F/GS/CHN/2008	GQ429255	2008	Gansu	B1a
520-03F/SD/CHN/2007	GQ429220	2007	Shandong	B1a
CNS68762/SAR/06	AM292451	2006	Malaysia	B1a
418/Toyama/2006	AB465402	2006	Japan	B1a
05.194.4135	FJ868280	2005	Australia	B1a
Siriraj07/TH/02Y95-2260	GQ184132AB634302	20051995	ThailandJapan	B1aB1a
CVA16/JN049/CHN/2018	MK357099	2018	Shandong	B1b
S5876/BJ/CHN/2018	MT553216	2018	Beijing	B1b
MY2018-05-CVA16	MW179119	2018	Sichuan	B1b
HLJ18-16/HLJ/North/CHN/2018-08-05	MT212012	2018	Heilongjiang	B1b
SD2018-YT231-CVA16-VP1	MT859144	2018	Shandong	B1b
CVA16-97_GD-CHN_2018-03	MW197349	2018	Guangdong	B1b
CVA16/SDJN100/CHN/2017	MH160028	2017	Shandong	B1b
SHCA2016-084	KX871438	2016	Shanghai	B1b
QH16-8/QH/West/CHN/2016-07-07	MT212020	2016	Qinghai	B1b
SHCA2016-112	KX871459	2016	Shanghai	B1b
CVA16/Shenzhen97/CHN/2016	MH003970	2016	Guangdong	B1b
CVA16/Shenzhen499/CHN/2015	MH003957	2015	Guangdong	B1b
CVA16/HuB/C15/2014	KY100914	2014	Hubei	B1b
C55-YN-CHN-2014	LC013399	2014	Yunnan	B1b
752-Henan-2013	KM260103	2013	Henan	B1b
998-Henan-2013	KM260082	2013	Henan	B1b
16-Henan-2013	KM260120	2013	Henan	B1b
GC13-049/NJ/CHN/2013	KP751473	2013	Jiangsu	B1b
S1870/BJ/CHN/2013	MT553144	2013	Beijing	B1b
12MM19_2012.05_JY	KP341909	2012	Jiangsu	B1b
SHCA2012-035	KX871273	2012	Shanghai	B1b
E052(Suzhou/2012)	MH491125	2012	Jiangsu	B1b
11MF56_2011.08_JJ	KP341818	2011	Jiangsu	B1b
lianyungang-1-2011	KR138313	2011	Jiangsu	B1b
Ningbo.CHN/113/2010	JQ315098	2010	Zhejiang	B1b
MAS03/AH/CHN/2010	JQ409497	2010	Anhui	B1b
QH0377T/QH/CHN/2008	GQ429271	2008	Qinghai	B1b
BJ271	JF317967	2008	Beijing	B1b
CF312044_FRA10	HE572997	2010	France	B1c
CF341014_FRA10	HE573002	2010	France	B1c
SB16087/SAR/05	AM292476	2005	Malaysia	B1c
CVA-16/21/CHN/GZ/2018	MT119447	2018	Guangdong	B1c
2680-Yamagata-2011	AB772007	2011	Japan	B1c
SB2239/SAR/00	AM292468	2000	Malaysia	B2
576/Toyama/1988	AB465368	1988	Japan	B2
S10432/SAR/98	AM292455	1998	Malaysia	B2
Y92-2861	AB634291	1992	Japan	B2

### Nucleotide sequence accession numbers

The nucleotide sequences used in this study were submitted in GenBank (https://www.ncbi.nlm.nih.gov/genbank/) under accession numbers OP373746-OP373967, which are shown in **Supplementary Table S1**.

## Results

### Epidemiological features

From January 1, 2010 to December 31, 2021, a total of 71,158 HFMD cases were reported in Taiyuan City. Mild to moderate symptoms were present in the vast majority of cases (99.94%, 71116/71158); 42 cases were classified as severe and five of these died. The annual average incidence rate of HFMD was 123.69 cases per 100,000 individuals (range: 50.29–219.25 per 100,000 individuals). The patients included 42,515 males and 28,643 females, with a mean male-to-female sex ratio of 1.47:1 (range:1.26:1 to 1.64:1). The annual average incidence in males was 144.41 per 100,000 individuals and that in females was 101.20 per 100,000 individuals. Overall, males had a significantly higher prevalence of HFMD than that in females (χ² = 2188.249, *P* < 0.001). (**Table 2**)

**Table 2:** Data of reported hand, foot, and mouth disease (HFMD) cases by gender, age group, and year.

Variable	2010	2011	2012	2013	2014	2015	2016	2017	2018	2019	2020	2021
**HFMD Reported Cases**												
Mild cases	7177	8226	9278	4353	10108	7221	4472	7400	4647	4423	1100	2711
Severe cases	2	8	18	0	7	0	0	0	1	1	0	0
Deaths	1	1	2	0	1	0	0	0	0	0	0	0
Total (Incidence rate/10^5^)	7180(170.76)	8235(190.20)	9298(209.27)	4353(95.80)	10116(219.25)	7221(153.00)	4472(92.17)	7400(148.49)	4648(90.87)	4424(84.56)	1100(20.68)	2711(50.29)
**Gender**												
Male (Incidence rate/10^5^)	4289(199.18)	4879(220.66)	5779(257.00)	2633(113.81)	5910(252.37)	4363(180.91)	2728(110.15)	4395(172.94)	2723(104.72)	2673(100.44)	633(23.19)	1510(54.86)
Female (Incidence rate/10^5^)	2891(140.93)	3356(158.40)	3519(160.36)	1720(77.12)	4206(185.11)	2858(123.84)	1744(73.43)	3005(123.041)	1925(76.55)	1751(68.12)	467(18.04)	1201(45.52)
Gender ratio	1.48	1.45	1.64	1.53	1.41	1.53	1.56	1.46	1.41	1.53	1.36	1.26
**Age Group (%)**												
<1	244(3.40%)	349(4.24%)	409(4.40%)	326(7.49%)	416(4.11%)	506(7.01%)	197(4.41%)	572(7.73%)	291(6.26%)	188(4.25%)	65(5.91%)	169(6.23%)
1-<2	1475(20.54%)	1643(19.95%)	2057(22.12%)	1234(28.35%)	2129(21.05%)	2033(28.15%)	915(20.46%)	1655(22.36%)	1135(24.42%)	786(17.77%)	204(18.55%)	610(22.50%)
2-<3	1453(20.24%)	1694(20.57%)	1824(19.62%)	778(17.87%)	1877(18.55%)	1312(18.17%)	810(18.11%)	1060(14.32%)	742(15.96%)	693(15.66%)	162(14.73%)	384(14.17%)
3-<4	1848(25.74%)	1997(24.25%)	2004(21.55%)	841(19.32%)	2273(22.47%)	1325(18.35%)	893(19.97%)	1620(21.89%)	760(16.35%)	910(20.57%)	244(22.18%)	414(15.27%)
4-<5	1090(15.18%)	1301(15.80%)	1439(15.48%)	563(12.93%)	1576(15.58%)	893(12.36%)	819(18.31%)	1110(15.00%)	711(15.30%)	623(14.08%)	169(15.36%)	439(16.19%)
5-<6	465(6.48%)	608(7.38%)	680(7.31%)	265(6.09%)	800(7.91%)	506(7.01%)	346(7.74%)	638(8.62%)	401(8.63%)	515(11.64%)	77(7.00%)	247(9.11%)
≧6	605(8.42%)	643(7.81%)	885(9.52%)	346(7.95%)	1045(10.33%)	646(8.95%)	492(11.00%)	745(10.07%)	608(13.08%)	709(16.03%)	179(16.27%)	448(16.53%)

From 2010 to 2021, most HFMD cases were observed in the age group of one to five years in Taiyuan, Shanxi Province [84.42% (60075/71158)], whereas incidence in children under one year of age (minimum age 1 day) was very low, and accounted for only 5.48%. Other details regarding age are shown in **Table 2**. The highest percentage of severe and fatal cases was observed in the age group of one–three years.

### Seasonal characteristics of HFMD

**Figure 1:**
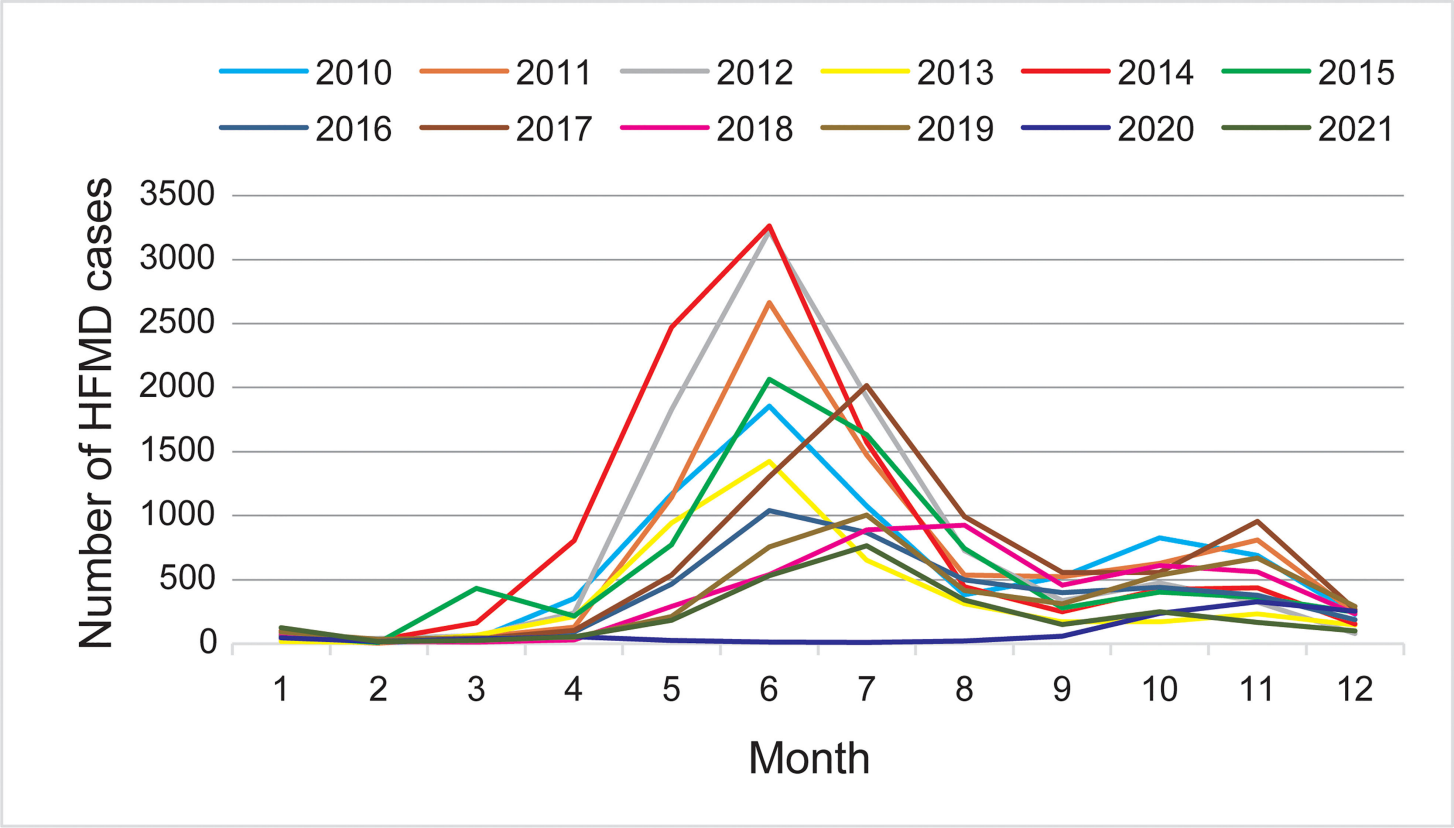
Monthly distribution of hand, foot, and mouth disease (HFMD) cases during 2010–2021 in Taiyuan.

Except being affected by the COVID-19 epidemic in 2020, the monthly distribution of HFMD cases showed two epidemic peaks; the main peak was from May to August and the second peak was from October to December. HFMD epidemics were sporadic from January to March each year, and began to increase in April, with a major epidemic peak from May to August; they declined in September, followed by a secondary peak from October to December. And the peak of cases showed a slight but obvious shift from June before (including) 2016 to July since 2017. During the COVID-19 pandemic, an epidemic peak of HFMD was only observed from October to December in 2020, which accounted for 74.33% of the annual cases (**Figure 1**).

### Pathogen spectrum of HFMD

**Figure 2:**
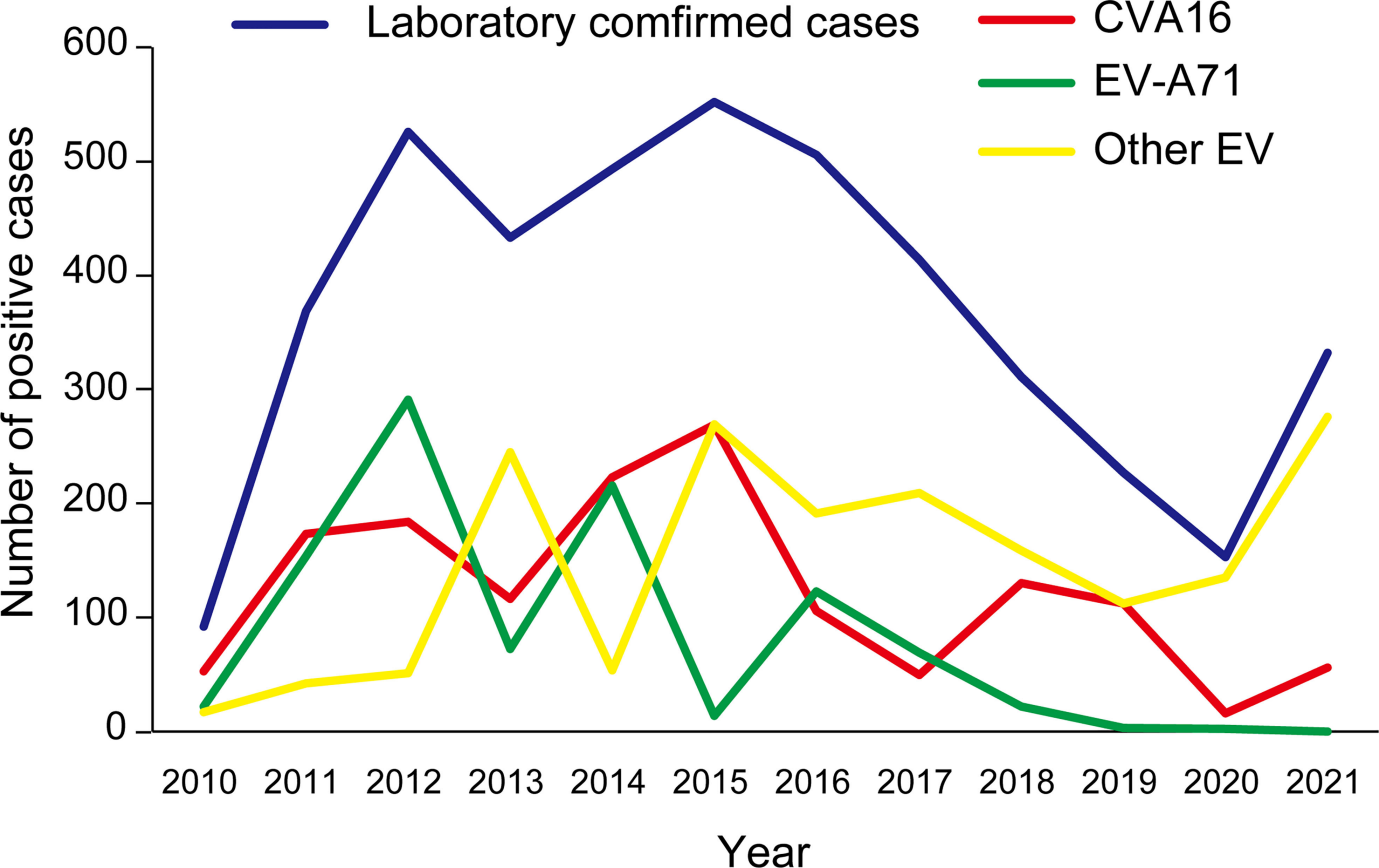
Genotype of viruses causing hand, foot, and mouth disease during 2010–2021 in Taiyuan.

Enterovirus nucleic acid was detected using real-time reverse transcription polymerase chain reaction in 6641 clinical specimens collected from patients with HFMD from 2010–2021; overall, 4236 EV-positive specimens were detected, including 988(23.32%) EV-A71, 1488(35.13%) CVA16, and 1760(41.55%) other enteroviruses (**Table S2**). The results showed that the two major pathogens causing HFMD before 2016 were EV-A71 and CVA16, although other EVs have also been reported. After 2016, HFMD cases caused by EV-A71 gradually decreased; however, CVA16 remains prevalent and has co-circulated with other EVs in Taiyuan (**Figure 2**).

### Phylogenetic analysis of CVA16 based on the *VP1* sequence

**Figure 3:**
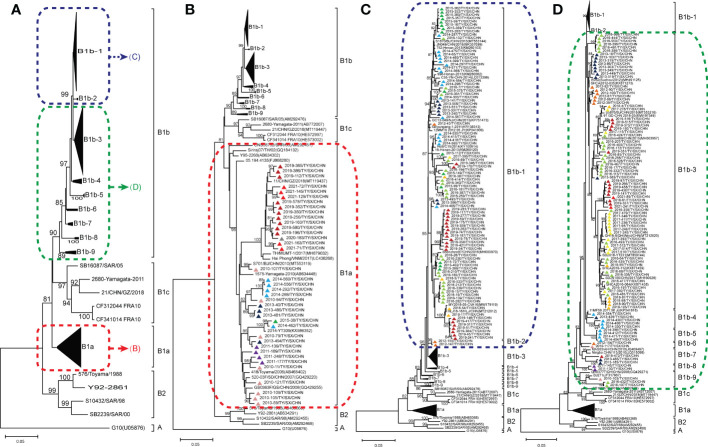
Phylogenetic analysis for subtyping 222 Coxsackievirus A16 (CVA16) strains in Taiyuan based on their VP1 sequences **(A)**; CVA16 subtype B1a **(B)**; CVA16 subtypes B1b-1, B1b-2 **(C)**; and CVA16 subtypes B1b-3, B1b-4, B1b-5, B1b-6, B1b-7, B1b-8, and B1b-9 **(D)**. Percentages of bootstrap values (1000 pseudo replicates) ≥ 80% are shown at the nodes. The CVA16 strains in Taiyuan are represented by triangles. Triangles of different colors represent different years. 2010 (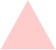) 2011 (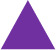) 2012 (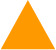) 2013 (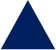) 2014 (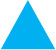) 2015 (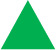) 2016 (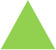) 2017 (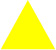) 2018 (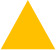) 2019 (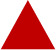) 2020 (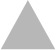) 2021(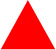).

To determine the genetic characteristics of CVA16 strains, we removed the identical sequences for the same month of the same year. Finally, a total of 273 CVA16 strains were used for phylogenetic analysis, including 222 CVA16 strains selected in this study and 51 other CVA16 reference strains obtained from GenBank. The 222 Taiyuan CVA16 strains in the phylogenetic tree were divided into two clades, belonging to the B1a and B1b evolutionary branches (**Figure 3A**). Among all strains, 184 were identified as subtype B1b and 38 as B1a. The 38 B1a strains were prevalent in 2010–2015 and in 2019–2021 (**Figure 3B**). The 184 B1b strains, were divided into two major prevalent branches, one prevalent in 2012–2019 and the other in 2016–2021; and the details of B1b are shown in **Figure 3C**, **D**.

Upon calculating the similarities in nucleotides and amino acids, the sequences of 362 strains of CVA16 were found to have high similarity with each other; the nucleotide similarity was 86.5–100% and the amino acid similarity was 96.9–100%. Further, the CVA16 strains showed 74.2–77.2% and 90.9–92.2% similarity in the nucleotide and amino acid sequence with the CVA16 prototype strain (G10 strains), respectively. Thirty-eight B1a CVA16 strains shared 90.5–99.8% and 97.6–100% similarity in nucleotide and amino acid sequences, respectively, whereas 184 B1b CVA16 strains shared 90.6–99.8% and 97.9–100% similarity in the nucleotide and amino acid sequence, respectively.

## Discussion

Based on the data from a twelve-year (2010–2021) surveillance of HFMD cases in Taiyuan city, our study provides a comprehensive landscape of the epidemiology of HFMD in Taiyuan City, Shanxi province, China. The results indicate that children aged 1–5 years accounted for most outbreaks, similar to reports from other regions in China and other countries (Chen et al., 2007; Momoki, 2009; Xing et al., 2014; Gui et al., 2015; Sun et al., 2017). These results suggest that children in this age group are at a high risk of enterovirus infection and should be targeted for HFMD control and prevention. More infections were found in males than in females. One explanation may be related to the extra outdoor activities by boys, including more frequent touching of unclean toys and facilities, which increases the level of exposure and the risk of the infection compared with that in girls. Further, the differential susceptibility of males and females at the level of host immune status, may also be responsible for this inconsistent sex ratio (Xing et al., 2014). Therefore, it is necessary to improve the hygiene status of children younger than 5 years of age to help reduce the risk of developing HFMD. Although HFMD cases have been reported throughout the year in Taiyuan City, the monthly distribution showed two peaks in HFMD epidemics, with the main peak between May and August, possibly because the virus spread is affected by increased activities of children during May and August of the year. The reason is unclear for the peak of HFMD cases shifted from before June 2016 (including 2016) to July 2017, it may be related to the sharp reduction in HFMD caused by EV-A71 following the introduction of the EV-A71 vaccine in 2016. A smaller peak occurs in November, probably because the cold season is conducive to the surviving of the virus. These results indicate epidemiological surveillance and prevention efforts should focus on the periods from May to August and from October to December.

Alternating outbreaks of CVA16 and EV-A71, considered to be main pathogens of HFMD, have occurred in East and Southeast Asia for more than 20 years (Li et al., 2005; Iwai et al., 2009). Outbreaks of HFMD with CVA16 have been reported in several cities in mainland China. However, long-term surveillance studies on CVA16 remain scarce (Zou et al., 2012; Zhu et al., 2013). In this 12-year surveillance study, the results showed an epidemic trend of both pathogens, from alternating with each other to CVA16 dominating over EV-A71. Before 2016, the two major pathogens causing HFMD were EV-A71 and CVA16, though other EVs have also been reported to cause HFMD. However, after administration of the EV-A71 inactivated vaccine, the pathogen spectrum of HFMD has changed to some extent, and the number of HFMD cases caused by EV-A71 has gradually decreased; however, CVA16 remains prevalent and has become one of the most important pathogens causing HFMD in Taiyuan City. Therefore, our study focused on the genetic characteristics of CVA16 to broaden our knowledge regarding this pathogen.

Numerous studies on CVA16 genotyping have shown that CVA16 can be divided into two genotypes: A and B (Perera et al., 2007; Zhang et al., 2010; Zhang et al., 2019). The prototype strain (G10/RSA/1951) is the only member of genotype A (Xu et al., 2018). Genotype B is divided into the subgenotypes B1 and B2, which are further divided into the evolutionary branches B1a, B1b, and B1c, and B2a, B2b, and B2c, respectively. Subgenotype B2 was first reported in Japan in 1981 and was not reported after 2000. Relatively, subgenotype B1 has been more common in recent HFMD outbreaks. CVA16 belonging to the evolutionary branches B1a and B1b were first isolated in 1995 and 1998, respectively, in Japan and became the predominant strains circulating globally since 2000. After 2000, subgenotype B1c was found in Malaysia, Russia, France, Japan, India, and other countries (Chen et al., 2013; Hassel et al., 2017; Gopalkrishna and Ganorkar, 2021). The first Chinese case of strain B1c (MT119447) was reported in Guangdong in 2018 (Xie et al., 2020).

In mainland China, most reported CVA16 strains belong to the B1a and B1b evolutionary branches (Chen et al., 2013). Our results showed that all CVA16 strains found in Taiyuan City belonged to the B1 subgenotype. Thirty-eight Taiyuan CVA16 strains belonged to the B1a evolutionary branch, responsible for two HFMD outbreaks being occurred from 2010 to 2015 and 2019 to 2021, respectively. This suggests that B1a disappeared after a period of prominence in Taiyuan City and became prevalent again in 2019. The other 184 Taiyuan CVA16 strains belonged to the B1b evolutionary branch, and were closely related to other domestic strains, such as Jiangsu, Henan, Shanghai, Shandong, and Guangdong. Except 2020, B1b was prevalent in other years. B1b was not detected in 2020; this may be attributed to the outbreak of COVID-19, which reduced the spread of viruses. The 184 strains belonging to the B1b evolutionary branch further showed two major prevalent branches, one in 2012–2019, and the other in 2016–2021. The remaining 21 B1b strains were prevalent in 2010–2014, 2016, and 2019. This indicated that although B1b was epidemic from 2010 to 2011, a new evolutionary branch appeared after 2012, which remained stable until 2019. During this period, new transmission chains emerged and have become prevalent since 2016. Thus, the two evolutionary branches of CVA16, B1a and B1b, coexisted and prevailed in Taiyuan from 2010 to 2021, with B1b as the dominant subtype. This observation is consistent with findings from other regions of China in recent years (Yin et al., 2014; Huang et al., 2015; Xia et al., 2016; Sun et al., 2017).

Our study has several limitations. First, the laboratory-confirmed cases in this study may have sampling bias, which may lead to further bias in our research results. Second, our study focused on the VP1 region of CVA16, which makes the genetic characterization imperfect; the further studies on the full-length genomic sequences of CVA16 are thus needed to illuminate the epidemiological and evolutionary dynamics of CVA16.

## Conclusions

This study highlights the epidemiological characteristics of HFMD, and main pathogens of HFMD cases reported in Taiyuan City, Shanxi province from 2010 to 2021, focusing on the genetic characteristics of CVA16, as well as on the coexistence and propagation of the B1a and B1b evolutionary branches. These findings add to the available genetic resources on CVA16 worldwide. The surveillance and research results of enteroviruses in various places indicate that the epidemic trend of each type of enterovirus is changing and varies from place to place. Thus, the HFMD pathogen spectrum should be determined in a timely manner to provide a scientific basis for HFMD prevention, control, and early warning.

## Data availability statement

The datasets presented in this study can be found in online repositories. The names of the repository/repositories and accession number(s) can be found below: GenBank (https://www.ncbi.nlm.nih.gov/genbank/) and the accession numbers are OP373746-OP373967.

## Ethics statement

This study did not involve human participants or human experimentation. The human materials used were throat swabs, fecal swabs, herpes fluid, and cerebrospinal fluid collected from patients with HFMD for public health purposes. Specimens were analyzed after informed and verbal consent obtained from the parents or guardians of the children. This study was approved by the Institutional Review Board and Human Research Ethics Committee of the Taiyuan Center for Disease Control and Prevention, and the methods were carried out in accordance with the approved

## Author contributions

JW and JG developed the original idea, drafted and reviewed the manuscript. YZ developed the original idea and contributed to the manuscript revision. ZC performed all the experiments, analyzed the data, and wrote the manuscript. HL engaged in the experimental operation and data analysis. JX, LZ and LG contributed to the specimens collection and provided technical support. ZZ, YS and ZH gathered and analyzed the patient data. All authors read and approved the final manuscript.

## Funding

This study was supported by a Science and Technology research project of the Shanxi Provincial Department of Health (Project No. 201302026); National Key Research and Development Program of China (Project No. 2021YFC2302003), and Natural Science Foundation of Beijing (Project No. L192014).

## Acknowledgments

The authors wish to thank county CDC employees in Taiyuan City who were responsible for the specimen collection process.

## Conflict of interest

The authors declare that the research was conducted in the absence of any commercial or financial relationships that could be construed as a potential conflict of interest.

## Publisher’s note

All claims expressed in this article are solely those of the authors and do not necessarily represent those of their affiliated organizations, or those of the publisher, the editors and the reviewers. Any product that may be evaluated in this article, or claim that may be made by its manufacturer, is not guaranteed or endorsed by the publisher.

## Supplementary material

Supplementary Table 1 GenBank accession numbers assigned for all the CVA16-Taiyuan sequences based on entire VP1 region sequenced in this study.

Supplementary Table 2 Etiological composition of laboratory-detected HFMD cases in Taiyuan City, 2010-2021.

## Abbreviations

HFMD, Hand, foot, and mouth disease; EV: Enterovirus; CVA16, Coxsackievirus A16; EV-A71, Enterovirus A71; CDC, Center for Disease Control and Prevention; Ct, Cycle threshold.
