# Supplementary material for: Epidemiology of hand, foot, and mouth disease and the genetic characteristics of Coxsackievirus A16 in Taiyuan, Shanxi, China from 2010 to 2021
Source: Front Cell Infect Microbiol. 2022 Nov 11;12:1040414. doi: 10.3389/fcimb.2022.1040414 (PMC9692002; doi:10.3389/fcimb.2022.1040414)
Supplement: Supplementary Table 1 — GenBank accession numbers assigned for all the CVA16-Taiyuan sequences based on entire VP1 region sequenced in this study. [file Table_1.docx]

Table S1 GenBank accession numbers assigned for all the CVA16-Taiyuan sequences based on entire VP1 region sequenced in this study.

| Coxsackievirus A16 (VP1) | | | |
| --- | --- | --- | --- |
| Accession Number | Strain | Accession Number | Strain |
| OP373746 | CVA16-2021-89/TY/SX/CHN | OP373857 | CVA16-2015-99/TY/SX/CHN |
| OP373747 | CVA16-2021-241/TY/SX/CHN | OP373858 | CVA16-2015-72/TY/SX/CHN |
| OP373748 | CVA16-2019-97/TY/SX/CHN | OP373859 | CVA16-2015-59/TY/SX/CHN |
| OP373749 | CVA16-2019-91/TY/SX/CHN | OP373860 | CVA16-2015-402/TY/SX/CHN |
| OP373750 | CVA16-2019-75/TY/SX/CHN | OP373861 | CVA16-2015-399/TY/SX/CHN |
| OP373751 | CVA16-2019-74/TY/SX/CHN | OP373862 | CVA16-2015-375/TY/SX/CHN |
| OP373752 | CVA16-2019-59/TY/SX/CHN | OP373863 | CVA16-2015-369/TY/SX/CHN |
| OP373753 | CVA16-2019-516/TY/SX/CHN | OP373864 | CVA16-2015-362/TY/SX/CHN |
| OP373754 | CVA16-2019-515/TY/SX/CHN | OP373865 | CVA16-2015-360/TY/SX/CHN |
| OP373755 | CVA16-2019-51/TY/SX/CHN | OP373866 | CVA16-2015-357/TY/SX/CHN |
| OP373756 | CVA16-2019-472/TY/SX/CHN | OP373867 | CVA16-2015-353/TY/SX/CHN |
| OP373757 | CVA16-2019-458/TY/SX/CHN | OP373868 | CVA16-2015-283/TY/SX/CHN |
| OP373758 | CVA16-2019-45/TY/SX/CHN | OP373869 | CVA16-2015-16/TY/SX/CHN |
| OP373759 | CVA16-2019-430/TY/SX/CHN | OP373870 | CVA16-2015-149/TY/SX/CHN |
| OP373760 | CVA16-2019-421/TY/SX/CHN | OP373871 | CVA16-2015-112/TY/SX/CHN |
| OP373761 | CVA16-2019-417/TY/SX/CHN | OP373872 | CVA16-2014-65/TY/SX/CHN |
| OP373762 | CVA16-2019-384/TY/SX/CHN | OP373873 | CVA16-2014-584/TY/SX/CHN |
| OP373763 | CVA16-2019-373/TY/SX/CHN | OP373874 | CVA16-2014-571/TY/SX/CHN |
| OP373764 | CVA16-2019-359/TY/SX/CHN | OP373875 | CVA16-2014-568/TY/SX/CHN |
| OP373765 | CVA16-2019-358/TY/SX/CHN | OP373876 | CVA16-2014-567/TY/SX/CHN |
| OP373766 | CVA16-2019-357/TY/SX/CHN | OP373877 | CVA16-2014-565/TY/SX/CHN |
| OP373767 | CVA16-2019-351/TY/SX/CHN | OP373878 | CVA16-2014-555/TY/SX/CHN |
| OP373768 | CVA16-2019-346/TY/SX/CHN | OP373879 | CVA16-2014-554/TY/SX/CHN |
| OP373769 | CVA16-2019-34/TY/SX/CHN | OP373880 | CVA16-2014-475/TY/SX/CHN |
| OP373770 | CVA16-2019-317/TY/SX/CHN | OP373881 | CVA16-2014-471/TY/SX/CHN |
| OP373771 | CVA16-2019-283/TY/SX/CHN | OP373882 | CVA16-2014-460/TY/SX/CHN |
| OP373772 | CVA16-2019-281/TY/SX/CHN | OP373883 | CVA16-2014-430/TY/SX/CHN |
| OP373773 | CVA16-2019-277/TY/SX/CHN | OP373884 | CVA16-2014-418/TY/SX/CHN |
| OP373774 | CVA16-2019-266/TY/SX/CHN | OP373885 | CVA16-2014-415/TY/SX/CHN |
| OP373775 | CVA16-2019-241/TY/SX/CHN | OP373886 | CVA16-2014-412/TY/SX/CHN |
| OP373776 | CVA16-2019-187/TY/SX/CHN | OP373887 | CVA16-2014-408/TY/SX/CHN |
| OP373777 | CVA16-2019-175/TY/SX/CHN | OP373888 | CVA16-2014-406/TY/SX/CHN |
| OP373778 | CVA16-2019-170/TY/SX/CHN | OP373889 | CVA16-2014-404/TY/SX/CHN |
| OP373779 | CVA16-2019-150/TY/SX/CHN | OP373890 | CVA16-2014-399/TY/SX/CHN |
| OP373780 | CVA16-2019-147/TY/SX/CHN | OP373891 | CVA16-2014-398/TY/SX/CHN |
| OP373781 | CVA16-2019-146/TY/SX/CHN | OP373892 | CVA16-2014-330/TY/SX/CHN |
| OP373782 | CVA16-2019-117/TY/SX/CHN | OP373893 | CVA16-2014-303/TY/SX/CHN |
| OP373783 | CVA16-2018-98/TY/SX/CHN | OP373894 | CVA16-2014-298/TY/SX/CHN |
| OP373784 | CVA16-2018-90/TY/SX/CHN | OP373895 | CVA16-2014-297/TY/SX/CHN |
| OP373785 | CVA16-2018-87/TY/SX/CHN | OP373896 | CVA16-2014-287/TY/SX/CHN |
| OP373786 | CVA16-2018-55/TY/SX/CHN | OP373897 | CVA16-2013-86/TY/SX/CHN |
| OP373787 | CVA16-2018-54/TY/SX/CHN | OP373898 | CVA16-2013-558/TY/SX/CHN |
| OP373788 | CVA16-2018-5/TY/SX/CHN | OP373899 | CVA16-2013-551/TY/SX/CHN |
| OP373789 | CVA16-2018-4/TY/SX/CHN | OP373900 | CVA16-2013-519/TY/SX/CHN |
| OP373790 | CVA16-2018-38/TY/SX/CHN | OP373901 | CVA16-2013-493/TY/SX/CHN |
| OP373791 | CVA16-2018-17/TY/SX/CHN | OP373902 | CVA16-2013-483/TY/SX/CHN |
| OP373792 | CVA16-2018-105/TY/SX/CHN | OP373903 | CVA16-2013-449/TY/SX/CHN |
| OP373793 | CVA16-2017-95/TY/SX/CHN | OP373904 | CVA16-2013-429/TY/SX/CHN |
| OP373794 | CVA16-2017-514/TY/SX/CHN | OP373905 | CVA16-2013-396/TY/SX/CHN |
| OP373795 | CVA16-2017-510/TY/SX/CHN | OP373906 | CVA16-2013-365/TY/SX/CHN |
| OP373796 | CVA16-2017-479/TY/SX/CHN | OP373907 | CVA16-2013-315/TY/SX/CHN |
| OP373797 | CVA16-2017-411/TY/SX/CHN | OP373908 | CVA16-2013-307/TY/SX/CHN |
| OP373798 | CVA16-2017-395/TY/SX/CHN | OP373909 | CVA16-2013-303/TY/SX/CHN |
| OP373799 | CVA16-2017-393/TY/SX/CHN | OP373910 | CVA16-2013-249/TY/SX/CHN |
| OP373800 | CVA16-2017-392/TY/SX/CHN | OP373911 | CVA16-2013-240/TY/SX/CHN |
| OP373801 | CVA16-2017-346/TY/SX/CHN | OP373912 | CVA16-2013-185/TY/SX/CHN |
| OP373802 | CVA16-2017-312/TY/SX/CHN | OP373913 | CVA16-2013-18/TY/SX/CHN |
| OP373803 | CVA16-2017-278/TY/SX/CHN | OP373914 | CVA16-2013-141/TY/SX/CHN |
| OP373804 | CVA16-2017-246/TY/SX/CHN | OP373915 | CVA16-2013-103/TY/SX/CHN |
| OP373805 | CVA16-2017-245/TY/SX/CHN | OP373916 | CVA16-2012-98/TY/SX/CHN |
| OP373806 | CVA16-2017-236/TY/SX/CHN | OP373917 | CVA16-2012-90/TY/SX/CHN |
| OP373807 | CVA16-2017-115/TY/SX/CHN | OP373918 | CVA16-2012-82/TY/SX/CHN |
| OP373808 | CVA16-2016-89/TY/SX/CHN | OP373919 | CVA16-2012-67/TY/SX/CHN |
| OP373809 | CVA16-2016-88/TY/SX/CHN | OP373920 | CVA16-2012-43/TY/SX/CHN |
| OP373810 | CVA16-2016-85/TY/SX/CHN | OP373921 | CVA16-2012-39/TY/SX/CHN |
| OP373811 | CVA16-2016-7/TY/SX/CHN | OP373922 | CVA16-2012-155/TY/SX/CHN |
| OP373812 | CVA16-2016-64/TY/SX/CHN | OP373923 | CVA16-2012-154/TY/SX/CHN |
| OP373813 | CVA16-2016-568/TY/SX/CHN | OP373924 | CVA16-2012-147/TY/SX/CHN |
| OP373814 | CVA16-2016-550/TY/SX/CHN | OP373925 | CVA16-2012-100/TY/SX/CHN |
| OP373815 | CVA16-2016-535/TY/SX/CHN | OP373926 | CVA16-2011-145/TY/SX/CHN |
| OP373816 | CVA16-2016-533/TY/SX/CHN | OP373927 | CVA16-2011-130/TY/SX/CHN |
| OP373817 | CVA16-2016-528/TY/SX/CHN | OP373928 | CVA16-2010-117/TY/SX/CHN |
| OP373818 | CVA16-2016-493/TY/SX/CHN | OP373929 | CVA16-2010-103/TY/SX/CHN |
| OP373819 | CVA16-2016-491/TY/SX/CHN | OP373930 | CVA16-2019-352/TY/SX/CHN |
| OP373820 | CVA16-2016-481/TY/SX/CHN | OP373931 | CVA16-2019-350/TY/SX/CHN |
| OP373821 | CVA16-2016-467/TY/SX/CHN | OP373932 | CVA16-2010-107/TY/SX/CHN |
| OP373822 | CVA16-2016-444/TY/SX/CHN | OP373933 | CVA16-2019-256/TY/SX/CHN |
| OP373823 | CVA16-2016-432/TY/SX/CHN | OP373934 | CVA16-2019-196/TY/SX/CHN |
| OP373824 | CVA16-2016-424/TY/SX/CHN | OP373935 | CVA16-2019-160/TY/SX/CHN |
| OP373825 | CVA16-2016-417/TY/SX/CHN | OP373936 | CVA16-2019-365/TY/SX/CHN |
| OP373826 | CVA16-2016-414/TY/SX/CHN | OP373937 | CVA16-2019-580/TY/SX/CHN |
| OP373827 | CVA16-2016-400/TY/SX/CHN | OP373938 | CVA16-2019-578/TY/SX/CHN |
| OP373828 | CVA16-2016-399/TY/SX/CHN | OP373939 | CVA16-2019-112/TY/SX/CHN |
| OP373829 | CVA16-2016-389/TY/SX/CHN | OP373940 | CVA16-2019-386/TY/SX/CHN |
| OP373830 | CVA16-2016-387/TY/SX/CHN | OP373941 | CVA16-2020-183/TY/SX/CHN |
| OP373831 | CVA16-2016-361/TY/SX/CHN | OP373942 | CVA16-2021-163/TY/SX/CHN |
| OP373832 | CVA16-2016-359/TY/SX/CHN | OP373943 | CVA16-2021-145/TY/SX/CHN |
| OP373833 | CVA16-2016-340/TY/SX/CHN | OP373944 | CVA16-2021-129/TY/SX/CHN |
| OP373834 | CVA16-2016-339/TY/SX/CHN | OP373945 | CVA16-2021-72/TY/SX/CHN |
| OP373835 | CVA16-2016-336/TY/SX/CHN | OP373946 | CVA16-2015-38/TY/SX/CHN |
| OP373836 | CVA16-2016-332/TY/SX/CHN | OP373947 | CVA16-2014-462/TY/SX/CHN |
| OP373837 | CVA16-2016-282/TY/SX/CHN | OP373948 | CVA16-2014-560/TY/SX/CHN |
| OP373838 | CVA16-2016-26/TY/SX/CHN | OP373949 | CVA16-2014-556/TY/SX/CHN |
| OP373839 | CVA16-2016-259/TY/SX/CHN | OP373950 | CVA16-2014-292/TY/SX/CHN |
| OP373840 | CVA16-2016-256/TY/SX/CHN | OP373951 | CVA16-2014-288/TY/SX/CHN |
| OP373841 | CVA16-2016-230/TY/SX/CHN | OP373952 | CVA16-2013-494/TY/SX/CHN |
| OP373842 | CVA16-2016-212/TY/SX/CHN | OP373953 | CVA16-2013-486/TY/SX/CHN |
| OP373843 | CVA16-2016-210/TY/SX/CHN | OP373954 | CVA16-2013-481/TY/SX/CHN |
| OP373844 | CVA16-2016-207/TY/SX/CHN | OP373955 | CVA16-2013-40/TY/SX/CHN |
| OP373845 | CVA16-2016-197/TY/SX/CHN | OP373956 | CVA16-2011-248/TY/SX/CHN |
| OP373846 | CVA16-2016-194/TY/SX/CHN | OP373957 | CVA16-2011-189/TY/SX/CHN |
| OP373847 | CVA16-2016-19/TY/SX/CHN | OP373958 | CVA16-2011-177/TY/SX/CHN |
| OP373848 | CVA16-2016-185/TY/SX/CHN | OP373959 | CVA16-2011-139/TY/SX/CHN |
| OP373849 | CVA16-2016-181/TY/SX/CHN | OP373960 | CVA16-2010-121/TY/SX/CHN |
| OP373850 | CVA16-2016-172/TY/SX/CHN | OP373961 | CVA16-2010-94/TY/SX/CHN |
| OP373851 | CVA16-2016-17/TY/SX/CHN | OP373962 | CVA16-2010-109/TY/SX/CHN |
| OP373852 | CVA16-2016-15/TY/SX/CHN | OP373963 | CVA16-2010-105/TY/SX/CHN |
| OP373853 | CVA16-2016-139/TY/SX/CHN | OP373964 | CVA16-2010-79/TY/SX/CHN |
| OP373854 | CVA16-2016-132/TY/SX/CHN | OP373965 | CVA16-2010-59/TY/SX/CHN |
| OP373855 | CVA16-2016-113/TY/SX/CHN | OP373966 | CVA16-2010-11/TY/SX/CHN |
| OP373856 | CVA16-2016-107/TY/SX/CHN | OP373967 | CVA16-2021-71/TY/SX/CHN |
