# Supplementary material for: Epidemiology of hand, foot, and mouth disease and the genetic characteristics of Coxsackievirus A16 in Taiyuan, Shanxi, China from 2010 to 2021
Source: Front Cell Infect Microbiol. 2022 Nov 11;12:1040414. doi: 10.3389/fcimb.2022.1040414 (PMC9692002; doi:10.3389/fcimb.2022.1040414)
Supplement: Supplementary Table 2 — Etiological composition of laboratory-detected HFMD cases in Taiyuan City, 2010-2021. [file Table_2.docx]

**Table S2. Etiological composition of laboratory-detected HFMD cases in Taiyuan City, 2010-2021**

| Year | Number of specimens | EV-A71 | CVA16 | Other EVs | EV-positive |
| --- | --- | --- | --- | --- | --- |
| 2010 | 146 | 22 | 53 | 17 | 92 |
| 2011 | 565 | 154 | 173 | 42 | 369 |
| 2012 | 667 | 291 | 184 | 51 | 526 |
| 2013 | 619 | 72 | 116 | 245 | 433 |
| 2014 | 653 | 216 | 223 | 54 | 493 |
| 2015 | 697 | 14 | 269 | 269 | 552 |
| 2016 | 586 | 123 | 106 | 191 | 420 |
| 2017 | 604 | 69 | 50 | 209 | 328 |
| 2018 | 616 | 22 | 130 | 159 | 311 |
| 2019 | 603 | 3 | 112 | 112 | 227 |
| 2020 | 292 | 2 | 16 | 135 | 153 |
| 2021 | 593 | 0 | 56 | 276 | 332 |
| total | 6641 | 988 | 1488 | 1760 | 4236 |
